# In vitro ruminal degradability of wheat straw cultivated with white-rot fungi adapted to mushroom farming conditions

**DOI:** 10.1038/s41598-023-34747-y

**Published:** 2023-05-13

**Authors:** Siriwan D. Martens, Vicki Wildner, Annette Zeyner, Olaf Steinhöfel

**Affiliations:** 1 Department of Animal Husbandry, Saxon State Office for Environment, Agriculture and Geology (LfULG), 04886 Köllitsch, Germany; 2 Institute of Agricultural and Nutritional Sciences, Martin Luther University Halle-Wittenberg, Halle (Saale), Germany

**Keywords:** Microbiology, Fungi

## Abstract

Biological treatment of cereal straw for ruminant nutrition purposes might present an environmentally friendly option of valorizing a widely available by-product of grain production for farming systems with low external input. Several strains of white-rot fungi have been selected in the past under mostly controlled laboratory conditions for their capacity of lignin degradation. The study adapted to conditions on farm for upscaling purposes. The development of the in vitro straw digestibility with two different moistening pre-treatments and inoculated with three different fungi species, namely *Pleurotus ostreatus*, *Ceriporiopsis subvermispora* and *Volvariella volvacea*, was determined up to 42 days of fermentation with five sampling times. The effect of physical straw pre-treatments on nutritional parameters was evaluated. The neutral detergent fiber digestibility (NDFD_30h_), enzymatically soluble organic substance (ELOS) and the gas production (Hohenheim Feed value Test, HFT) as indicators for in vitro ruminal degradability decreased over time independent of the fungus: HFT, ELOS and NDFD_30h_ by up to 50, 35 and 30% of the original straw. Remoistening and autoclaving the straw increased the gas production significantly by 2.6 mL/200 g dry matter (DM), and ELOS and NDFD_30h_ by 45 and 51 g/kg DM compared to the original straw (34.9 mL/200 mg DM, 342 g/kg DM, 313 g/kg NDF).

## Introduction

Lack of roughage for ruminants due to drought in parts of Europe in 2018–2020 has reactivated the search for alternative fiber sources to forage.

Cereal straw is widely available. In Germany alone, more than 8 Mio t dry matter (DM) are recycled yearly without further use^1^. However, the main obstacle for its extensive use in animal feeding is its low digestibility due to the high lignin content and its strong bonds in lignin- carbohydrate-complexes^2^.

After a series of investigations in chemical straw treatment to enhance digestibility in Germany and elsewhere^3–6^, since the 1990s more emphasis was placed on biological treatment using white-rot fungi. Various studies have proved that some species and strains are able to degrade lignin under certain conditions^7–11^. For example, *Ceriporiopsis subvermispora* (*C.s.*) delignified wheat straw and oak wood chips during the first 5 weeks of treatment in a laboratory study^12^. *Pleurotus ostreatus* (*P.o.*) expressed ligninolytic peroxidase^13^, and changed the ratio of the lignin polymers while increasing digestibility^14^. Banana leaves inoculated with *Volvariella volvacea* (*V.v.*) decreased in acid detergent fiber (ADF) and lignin (ADL)^15^. Despite the numerous publications, to date, to our knowledge no or at least few practical implementations have taken place on a larger scale on farm.

The aim of our study was to test selected fungal strains of *P.o., C.s.* and *V.v.* on their efficacy to enhance ruminal digestibility in vitro, to question the effects of the pretreatments and to identify whether they could be applied under practical farm conditions for example in Germany.

There exist different and contradictory statements with respect to the optimal fermentation time. Van Kuijk et al.^12^ found an optimal lignin degradation after 5 weeks with *C.s.*, while Owen et al.^16^ recommended a maximum fermentation time of 6–8 days in general to limit organic matter losses. Thus, samples were obtained during a time course for analysis.

## Materials and methods

### Statement on the use of plants

All local and national guidelines and legislation were adhered to when using agricultural cereal crops in the study.

Winter wheat (*Triticum aestivum* Linnaeus) straw without visible fungal infestation was harvested in July 2018 in Köllitsch (51.5° latitude, 13.1° longitude), Northern Saxony, Germany, and stored as square bales in a dry ambient. It was obtained from the Agricultural Teaching and Testing Station of the Saxon State Office for Environment, Agriculture and Geology and had been cultivated within the agricultural production. It had a chopping length of 7–10 cm.

In this article, four trials, performed in 2019, are described: three on fungal treatments (two with soaked and drained straw and two with only remoistened straw) (Table 1) and one trial on physical treatment.

**Table 1:** Biological treatments in the different trials.

		Trial 1-soak and drain	Trial 2-remoist and *V.v.*	Trial 3-remoist and *C.s./P.o.*
Inoculant	Strains	*C. subvermispora CBS 347.63, P. ostreatus CBS 411.71 & PO93*	*V. volvacea* DSM 6190	*C. subvermispora CBS 347.63, P. ostreatus CBS 411.71*
	Growth medium	Wheat grains	Wheat grains	Wheat grains
Straw	Soaking	Abundant H_2_O	2.7 L H_2_O/kg straw (→ 25% target DM)	2.7 L H_2_O/kg straw (→ 25% target DM)
	Draining	Yes	No	No
Storage	Ambient °C	21 °C	24 °C	23 °C
Sampling	After … d	0, 7, 14, 21, 28, 25, 42	0, 7, 14, 21, 28	0, 5, 7, 10, 14

### Biological treatment

An overview of the biological treatments is given in Table 1.

#### Preparation of inoculum

Grain spawn was produced using wheat grain inoculated with *Ceriporiopsis subvermispora* CBS 347.63, *Pleurotus ostreatus* CBS 411.71 and PO93 resp., *Volvariella volvacea* DSM 6190, incubated at 24 °C (30 °C in the case of *V. volvacea*) for 8–14 days as described in Martens et al.^17^.

#### Solid state fermentation of wheat straw

##### Trial 1—soaked and drained straw

Wheat straw was soaked in abundant tap water for 20–24 h, i.e. submerged, and drained afterwards for 2–3 h. This “drained straw” was then inoculated with grain spawn and incubated at 21 °C for a maximum of 6 weeks as described in Martens et al.^17^. See also Table 1. All samples were weighed at day 0 and were removed in triplicates in weekly intervals. Then they were weighed again. Besides the weight, the dry matter (DM) content and the pH value were determined. The straw was visually examined. Samples were frozen at − 20 °C for further analysis.

##### Trial 2 and 3—remoistened straw

A defined volume of water was added to the wheat straw. In this way, a target DM of 250 g/kg was achieved. After 22 h for absorbing the water and turning around in the meantime the remoistened straw was inoculated with grain spawn as described in Martens et al.^17^. See also Table 1.

Samples of around 1200 g were stored aerobically in perforated bags at 23–24 °C (^17^ for details) and removed in quadruplicates in weekly intervals in Trial 2 and after 0, 5, 7, 10 and 14 days in Trial 3. All samples were weighed at day 0 and when removed for analysis, the DM content and the pH value were determined. The straw was visually examined. Samples were frozen at − 20 °C for further analysis.

### Physical treatment

In a second step, after evaluating the analytical results of the first trials of the biological treatments, the influence of soaking plus draining or remoistening only, and autoclaving, on the nutrient content of straw was tested. Straw was packed in nylon bags. Treatments in triplicates:

A. Control (air dry straw)
B. Tap water (2680 ml/kg air dry straw) was added to the bags in a plastic tub, bags were turned over after some hours. After 20 h the content of the bags was loosely filled in the tub to soak the remaining water for one more hour.
C. As B. After that, loose straw was filled in a cage to autoclave at 121 °C for 20 min.
D. Abundant tap water was filled in buckets with nylon bags filled with straw. After 20 h of soaking, the bags were hanged to drain for 3 h.
E. As D. After that, loose straw was filled in a cage to autoclave at 121 °C for 20 min.

The samples were chemically analyzed.

### Chemical analysis

Samples of untreated and treated straw were analyzed for DM, crude ash, neutral detergent fiber assayed with a heat stable amylase and expressed exclusive of residual ash (aNDFom), acid detergent fiber expressed exclusive of residual ash (ADFom), acid detergent lignin (ADL), ether extract (EE), crude protein, enzymatically soluble organic substance (ELOS), gas production according to the Hohenheim Feed value Test (HFT) (all parameters according to VDLUFA^18^ and NDF digestibility (NDFD_30h_)^19^.

The following parameters were calculated for the exclusively physically treated samples: digestible aNDFom (g/kg DM) = NDFD_30h_ (in %)/100 * aNDFom (g/kg DM), indigestible aNDFom = aNDFom (g/kg DM) – digestible aNDFom (g/kg DM), Non-fiber carbohydrates (NFC) = (1000-(aNDFom + CP + EE + ash)), cellulose = (ADFom-ADL), the ratio ADL/ADFom as indicator of degree of lignification, total digestible nutrients TDN_grass_ = (NFC * 0.98) + (CP * 0.87) + (FA * 0.97 * 2.25) + (NDF * 0.93 *  (22.7 + 0.664 * NDFD_30h_)/100) − 10 (in % of DM, FA fatty acids = ether extract – 1; equation for grass according to Moore and Undersander^20^; NDFD_48h_ replaced with NDFD_30h_), estimated dry matter intake (DMI_grass_) = − 2.318 + 0.442 * CP − 0.01 * CP^2^ – 0.0638 * TDN + 0.000922 * TDN^2^ + 0.18 * ADFom − 0.00196*ADF^2^ – 0.00529 * CP * ADFom (for grass: Moore and Kunkle^21^), relative forage quality RFQ = (DMI_grass_, % of BW)*TDN_grass_, % of DM)/1.23 (Undersander and Moore^22^).

### Statistical analysis

For the biological treatments, the following effects on in vitro digestibility were tested:

1st trial (soaked and drained straw):

$${\text{Y}}_{{{\text{ij}}}} = \upmu \, + \,{\text{Fungus}}_{{\text{i}}} \, + \,{\text{Time}}_{{\text{j}}} \, + \,{\text{Fungus }}*{\text{ Time}}_{{{\text{ij}}}} \, + \,\varepsilon_{{{\text{ij}}}}$$

where µ = general mean, i = 1, 2, 3 (*C. subvermispora, P. ostreatus* (2 strains)), j = 1, 2, 3, …, 7 (0 d, 7 d, 14 d, 21 d, 28 d, 35 d, 42 d fermentation time), ε_ij_ = residual error

2nd and 3rd trial (remoistened straw):

$${\text{Y}}_{{{\text{ij}}}} = \upmu \, + \,{\text{Fungus}}_{{\text{i}}} \, + \,{\text{Time}}_{{\text{j}}} \, + \,{\text{Fungus }}*{\text{ Time}}_{{{\text{ij}}}} \, + \,\varepsilon_{{{\text{ij}}}}$$

where µ = general mean, i = 1, 2, 3 (*C. subvermispora, P. ostreatus* PO93, *V. volvacea*), j = 1, 2, 3, …, 5 (0 d, (5 d,) 7 d, (10 d,) 14 d, (21 d, 28 d) fermentation time), ε_ij_ = residual error

The software IBM^®^ SPSS^®^ Statistics (Version 19, SPSS, Inc., IBM Company©) was used. Variance analysis using the procedures univariate and multivariate was performed for the treatments after the respective storage time, while the posthoc Tukey test comprised the original straw values. Linear regression analysis was performed for various digestibility indicators, calculating the Pearson correlation.

For the physical treatments a two factorial model with fixed effects and interactions was applied using SAS^®^ (Version 9.4 TS Level 1M7, SAS Institute Inc., Cary, NC, USA, 2020):

$${\text{Y}}_{{{\text{ijkl}}}} = {\text{Wetting}}_{{\text{i}}} \, + \,{\text{Autoclaving}}_{{\text{j}}} \, + \,{\text{Wetting }}*{\text{ Autoclaving}}_{{{\text{ij}}}} \, + \,\varepsilon_{{{\text{ijk}}}}$$

where i = 1, 2 (remoistening, soaking + draining), j = 1, 2 (no, yes), ε_ijk_ = residual error

## Results

### DM losses

From the mere physical treatments, it was calculated that soaking in abundant water plus draining resulted in an average loss of 90.6 g/kg DM, while straw remoistened with a defined amount of water lost only 3.5 g/kg DM.

**Figure 1:**
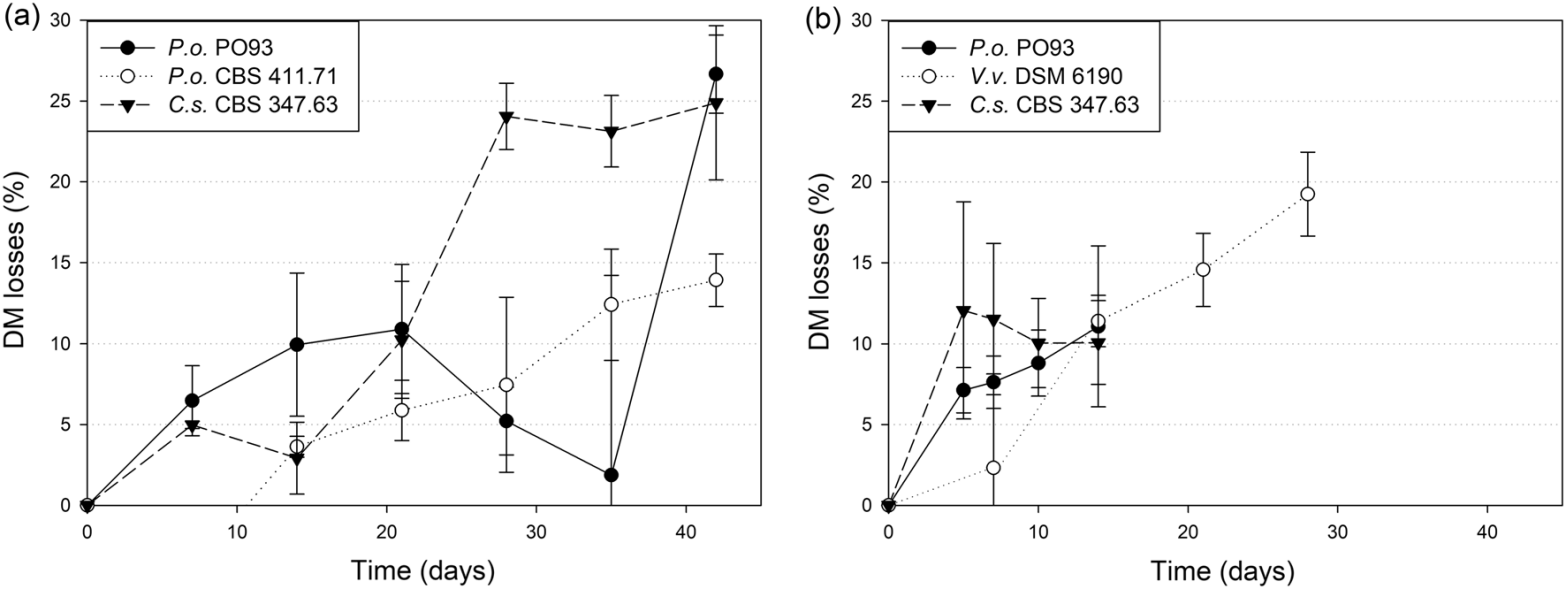
Dry matter losses calculated from the start of incubation: (**a**) drained straw inoculated with two strains of *Pleurotus ostreatus* (*P.o.*) and *Ceriporiopsis subvermispora* (*C.s*.), (**b**) remoistened straw inoculated with *Pleurotus ostreatus* (*P.o*.), *Volvariella volvacea* (*V.v*.) and *Ceriporiopsis subvermispora* (*C.s*.).

Within 14 days of fermentation the remoistened treatments lost around 108 g/kg DM (Trial 2 and 3), which was similar to the drained straw inoculated with PO93 (Trial 1) (excluding losses by draining) (Fig. 1a,b). Losses of the drained *C. subvermispora* treatment were comparably low at that point (29 g/kg DM), but increased to 241 g/kg by day 28 (Fig. 1a). A similar level of the drained treatment with PO93 was achieved only at day 42, while with *P. ostreatus* CBS 411.71 losses were lowest (139 g/kg at day 42). *V. volvacea* increased DM losses almost linearly from day 7 to 28 in the remoistened straw (y = 0.725x − 0.642, R^2^ = 0.97, *p* = 0.002) to 192 g/kg DM at day 28 (Fig. 1b).

### Changes in the chemical composition

#### Physical treatment

The results of chemical composition of straw following physical treatment are presented first (Tables 2, 3, 4). The interaction of type of wetting and autoclaving or not was significant for ADFom, whose content increased both by soaking plus draining and by autoclaving (Table 2). Parameters resulting from calculations including ADFom were equally affected. Most of the parameters were influenced by the type of wetting (Table 3). Draining led to higher EE, aNDFom and ADL contents while NDFD_30h_ and ELOS were reduced compared to remoistening. Autoclaving enhanced gas production slightly and increased ELOS (Table 4). When compared to the original straw, remoistening plus autoclaving increased the in vitro digestibility in terms of NDFD_30h_, gas production and ELOS.

**Table 2:** Chemical composition after physical treatment of wheat straw – interaction of wetting and autoclaving.

Wetting	Dry	Remoistened	Remoistened	Drained	Drained	SEM	p-value	Autoclaving	W*A
Autoclaving	No	No	Yes	No	Yes				
n	6	3	4	6	5		Wetting		
ADFom [g/kg DM]	408	443^c^	449^c^	469^b^	494^a^	2.291	< 0.001	< 0.001	0.015
Ratios									
Cellulose/ADL	6.99	8.52^a^	6.57^b^	6.44^b^	6.35^b^	0.142	0.001	0.002	0.005
ADL/ADFom	0.126	0.105^b^	0.132^a^	0.135^a^	0.137^a^	0.0024	0.005	0.017	0.038

ADFom acid detergent fibre exclusive of residual ash, ADL acid detergent lignin. SEM: standard error of the mean. Variance analysis excluding the untreated control. Treatments with different letters are significantly different (t-test, p < 0.05).

**Table 3:** Chemical composition after physical treatment of wheat straw—effect of wetting.

Wetting	Dry	Remoistened	Drained	SEM	p-value
Autoclaving	No	Mean	Mean		Wetting
Crude ash [g/kg DM]	78.5	85.3^a^	56.5^b^	0.601	< 0.001
Crude protein [g/kg DM]	41.5	36.8^a^	30.9^b^	1.249	0.006
EE [g/kg DM]	7.38	8.22^b^	12.2^a^	0.368	< 0.001
aNDFom [g/kg DM]	754	791^b^	834^a^	3.005	< 0.001
ADL [g/kg DM]	51.6	52.9^b^	65.7^a^	1.152	< 0.001
NFC [g/kg DM]	118	91.3^a^	66.6^b^	3.407	0.010
Ratios					
dig/indig aNDFom	0.430	0.526^a^	0.436^b^	0.0110	0.007
Calculated forage quality					
TDN [g/kg DM]	455	456^a^	438^b^	2.267	0.008
RFQ	56.6	53.5^a^	44.91^b^	0.822	< 0.001
DMI [g/kg BW]	15.3	14.4^a^	12.7^b^	0.019	< 0.001
In vitro digestibility					
NDFD_30h_ [g/kg NDF]	300	342^a^	303^b^	0.505	0.009
ELOS [g/kg DM]	348	378^a^	354^b^	0.386	0.006

EE ester extract, aNDFom amylase treated neutral detergent fibre exclusive of residual ash, ADL acid detergent lignin, NFC non-fibre carbohydrates, dig/indig digestible/indigestible ratio, TDN total digestible nutrients, RFQ relative forage quality index, DMI dry matter intake, BW body weight, NDFD_30h_ NDF digestibility after 30 h of incubation. SEM: standard error of the mean. Variance analysis excluding the untreated control. Treatments with different letters are significantly different (t-test, p < 0.05).

**Table 4:** Chemical composition after physical treatment of wheat straw – effect of autoclaving.

Wetting	Dry	Mean	Mean	SEM	p-value
Autoclaving	No	No	Yes		Autoclaving
Crude ash [g/kg DM]	78.5	72.7^a^	69.1^b^	0.601	0.034
Crude protein [g/kg DM]	41.5	36.6^a^	31.1^b^	1.249	0.009
ADL [g/kg DM]	51.6	54.8^b^	63.8^a^	1.152	0.005
Calculated forage quality					
RFQ	56.6	50.8^a^	47.6^b^	0.822	0.042
DMI [g/kg BW]	15.3	13.8^a^	12.9^b^	0.019	0.004
In vitro digestibility					
HFT [ml/200 mg DM]	33.0	34.4^b^	35.5^a^	0.238	0.034
ELOS [g/kg DM]	348	354^b^	378^a^	0.386	0.006

ADL acid detergent lignin, RFQ relative forage quality index, DMI dry matter intake, BW body weight, HFT gas production (Hohenheim feed value test), ELOS enzymatically soluble organic substance. SEM: standard error of the mean. Variance analysis excluding the untreated control. Treatments with different letters are significantly different (t-test, p < 0.05).

#### Biological treatment

**Figure 2:**
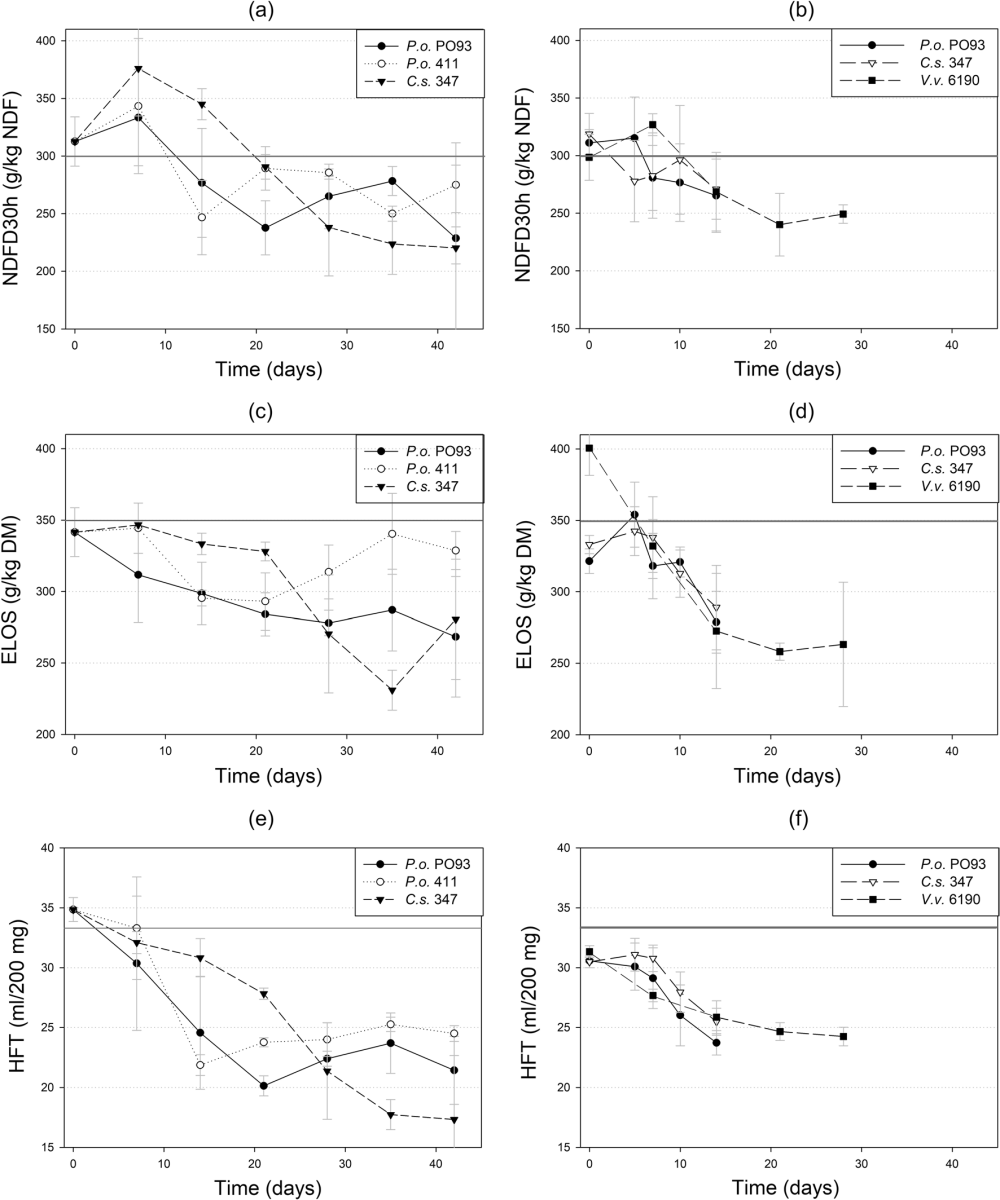
Changes over time of in vitro digestibility of wheat straw inoculated with different fungal strains. Left: drained straw inoculated with two strains of *Pleurotus ostreatus* (*P.o*.) and *Ceriporiopsis subvermispora* (*C.s*.), right: remoistened straw inoculated with *Pleurotus ostreatus* (*P.o*.), *Volvariella volvacea* (*V.v*.) and *Ceriporiopsis subvermispora* (*C.s*.). NDFD30h neutral detergent fibre digestibility, ELOS enzymatically soluble organic substance, HFT gas production (Hohenheim Feed value Test). Grey solid line: baseline from original untreated straw. Error bars represent the standard deviation SD.

There was a significant interaction of fungal strain and storage time of the drained straw for all three indicators of in vitro digestibility (Table 5). In the remoistened treatments, these indicators showed a uniform decrease starting in the second week and were less influenced by the fungal strain (Fig. 2). Although numerically higher, the NDFD_30h_ did not increase significantly during the first week after inoculation, both in the drained and the remoistened treatments (Fig. 2a,b). Instead, it decreased in the course of time. Comparing drained and remoistened straw inoculated with *P. ostreatus* PO93 and *C. subvermispora* after 7 and 14 days, only NDFD_30h_ of *C. subvermispora* in the drained straw was significantly higher than its counterpart in the remoistened straw. ELOS and HFT values were comparable within the two fungal strains at the same time. At no time either of the in vitro digestibility parameters was improved compared to the starting point or the dry control straw (Fig. 2, grey baseline).

**Table 5:** Significance of effects on in vitro digestibility in drained or remoistened straw inoculated with different fungi.

*p*	NDFD_30h_	ELOS	HFT	NDFD30h	ELOS	HFT
	Drained (Trial 1)			Remoistened (Trial 2 and 3)		
Fungal strain	ns	*	*	ns	*	*
Storage duration	***	**	***	**	***	***
Strain*duration	*	**	***	ns	**	ns

NDFD_30h_ neutral detergent fibre digestibility, ELOS enzymatically soluble organic substance, HFT gas production (Hohenheim Feed value Test).

## Discussion

Solid state fermentation of straw with basidiomycetes is an approach of valorizing field crop residues either for ruminant nutrition^10^ or for human mushroom consumption^23^ or biofuel production. Moisture, temperature, indigenous microflora are some factors, which influence both the growth of the fungi^24^, but also the nutritional composition and digestibility of the straw post-harvest^25^. To make uptake on farm likely, all processing steps have to be considered and minimized if possible. The same applies to losses from the field to the feeding trough.

Most of the studies on straw treatment with fungi soak the straw for several hours or days in abundant water and drain it then for several hours^11,26,27^. The presented study started with this following the recommendations for hobby mushroom cultivation^28^. However, in the chemical analysis it was realized that most of the fermentable carbohydrates got lost and that the lignin concentration increased by around 23%^17^ with decreasing digestibility at the same time. That is why the pre-treatment was changed to watering the straw with a limited volume of water to get a dry matter of approximately 25%. According to Streeter et al.^29^ a higher DM content (50%) was recommendable for incubation, which is in contrast to the observations by Abdullah et al.^30^ who recommended 80% moisture content for optimal fungal growth.

A second point in the pre-treatment is autoclaving, which is a common practice in laboratory studies (e.g.^31,32^). To make practical uptake on-farm more likely, this approach was abandoned.

To evaluate the effect of the physical pre-treatment a separate trial was performed. Remoistening plus autoclaving had the clearest effect on digestibility increase. Most advantages were seen in NDFD_30h_ (+ 17%) and ELOS (+ 12%). High-pressure steam treatment resulted in higher digestibility of DM and cell wall constituents with different roughages^33^, and even hot water at pH 4–7 had an effect by removing lignin and hemicellulose^34^.

The relation of cellulose/ADL was highest in the remoistened straw without autoclaving. However, this parameter was obviously less related to digestibility in contrast to the findings of Nayan et al.^11^, who determined a correlation of r = 0.64 between (cellulose + hemicellulose)/ADL and IVGP (in vitro gas production). The ratio of ADL/ADFom showed also no clear relationship to digestibility. The calculated forage quality parameters (TDN, RFQ, DMI) were similar between the remoistened and the original straw while they were lower for the drained treatments. This is linked to the formulae as they include both NFC contents and NDFD_30h_. As NFC contents were significantly reduced by almost half due to leaching this had a major influence on the parameters. However, ELOS remained on the same level as the original straw.

In contrast to these findings, no digestibility increase was found in the fermented straw although NDFD_30h_ was numerically higher within the first 7 days on average. That was a reason for shortening the fermentation period to 14 days in Trial 3 and having a look at closer sampling intervals.

In the presented trials, the same strain of *C. subvermispora* (CBS 347.63) was used as in other experiments performed in Wageningen. In those studies, the IVGP increased during a 7-week period of SSF based on drained and autoclaved wheat straw by around 30% although it was not compared to the original dry straw^11,27^. In any case, no increase in IVGP (HFT) was observed under the non-sterile conditions of the presented study. Decrease in digestibility in these trials went along with increase in lignin concentration by up to 42 g ADL/kg DM^17^. Nayan et al.^35^ suspected a problem in the ADL analyses when handling mushrooms as they observed increased IVGP by 28–48% despite high ADL values. However, gas production did not increase in the presented trials.

The *P. ostreatus* strain CBS 411.71 was earlier employed in an experiment for bioethanol production from wheat straw^36^. There, after 14 and 28 days, it improved enzymatic digestibility, increasing (hemi)cellulose digestibility from 35 to 55%. However, fermentable sugar yields were comparably low.

The generally observed decreasing digestibility in the presented trials might also be related to the lacking autoclaving in terms of sterilization prior to inoculation as the competing epiphytic microflora might have consumed the nutrients released by the white rot fungi similar to the observation made by Lang et al.^37^. Or fungal degradation was inhibited by an effect of competition with the native microflora^38,39^. In any case, Streeter et al.^29^ stated that autoclaving in his small samples sizes was not necessary. Tuyen et al.^40^ compared the gas production of straw inoculated with different fungi for up to 7 weeks to only autoclaved wheat straw (control). On day 21, out of 6 fungal species, *C. subvermispora* and *L. edodes* showed a higher gas production, on day 35, *P. eryngii* went beyond the control. However, the other species did not surpass the control line. *V. volvacea* inoculated straw declined linearly in IVGP from day 21 to day 49 of incubation^40^, which was more comparable to our observation.

For upscaling, Zadrazil et al.^7^ worked with non-sterile culture conditions. However, the author did not present digestibility differences compared to sterile conditions. Also Rai et al.^41^ seem to have worked without autoclaving using *Coprinus fimetarius* in rice straw and obtained results in feeding trials with goats in India, which were comparable to urea treated straw.

The temperature optimum for growth and metabolism differs from species to species and strain to strain. The temperature range for incubation from 21 to 24 °C applied in the presented study is similar to the one reported by Nayan et al.^27^, van Kuijk et al.^12^ and Fazaeli et al.^42^, although it is lower for *V. volvacea* compared to Belewu and Belewu^15^ with 35 °C for example. In any case, growth was observed for all inoculated strains, both under acidic and alkaline conditions^17^.

The treatment of draining and soaking alone led to about 10% DM losses and even higher losses in NFC concentration. Another 10% DM were lost within 14 days of incubation independent from the pre-treatment. Some studies quantify the different losses. With fungi of the genus *Ionotus* about 24% DM losses were observed after 30 days of incubation in wheat straw^43^. Zuo et al.^44^ found up to 50% DM loss with *Pleurotos chrysosporium* in maize stover after 28 days, which was relatively high compared to other findings^14,45^. Although a certain increase in in vitro DM digestibility was achieved when incubating wheat straw with *P. ostreatus* and *Erwinia carotovora*, DM losses of 52% question the loss of 69% lignin which led to a slightly higher IVDMD in another study^46^. Besides, DM loss did not necessarily go along with the desired loss of lignin^11,43^ and/or increase of digestibility as shown in the physical treatments here.

Some authors recommended a short fermentation period of 6–15 days to minimize losses^16,41,47,48^ and to increase nutrient intake in vivo^41^. Besides, some white rot fungi have a high initial selectivity for lignin^49^. Shrivastava et al.^50^ even found the highest in vitro OM digestibility after 5 days of SSF. That is one reason why in Trial 3 the fermentation time and the sampling intervals were reduced. However, it is in conflict with the different potential delignification phases described by Zadrazil et al.^7,25^ and van Kuijk et al.^9^.

## Conclusions

The study emphasizes the complexity of solid state fermentation with the purpose of ruminant nutrition. None of the tested fungal strains was able to improve the in vitro ruminal straw degradability under the given conditions. Pre-treatment gains the more importance the more it comes to upscaling. Moistening enables the fibrous complex to swell and thus be more easily attacked by the ruminal microbiome. As autoclaving is rather unrealistic for on-farm application, simple physical treatments such as the use of hot process water should be focused as a potential economical option to enhance ruminal fermentability of lignocellulosic materials.

## Data Availability

The datasets generated during the presented study are available from the corresponding author on reasonable request.
